# Turn-on fluorescent nanoprobe for ATP detection based on DNA-templated silver nanoclusters†

**DOI:** 10.1039/d3ra07077h

**Published:** 2024-02-13

**Authors:** Yuxia Li, Zeting Meng, Yating Liu, Baozhu Zhang

**Affiliations:** a Department of Chemistry and Chemical Engineering, Jinzhong University Yuci 030619 P. R. China zhangbaozhu518@126.com

## Abstract

A turn-on fluorescence nanoprobe was constructed for the determination of adenosine 5′-triphosphate (ATP) based on DNA-templated silver nanoclusters (DNA-AgNCs). The significant enhancement fluorescence intensity of DNA-AgNCs in the presence of ATP is due to the high special binding affinity between ATP and the aptamer, resulting in the environment of DNA-AgNCs with darkish fluorescence lying at one terminus of DNA slightly altering owing to the change of ATP aptamer conformation. A good linear range runs from 9 to 24 mM with a satisfactory detection limit of 3 μM. Furthermore, the proposed nanoprobe exhibited good performance for ATP detection in diluted fetal bovine serum.

## Abstract 2

A novel turn-on fluorescent nanoprobe was constructed based on the alteration of the aptamer conformation induced by target recognition and turn-on fluorescence due to alteration of its environment of DNA-templated silver nanoclusters (DNA-AgNCs).

## Graphical abstract

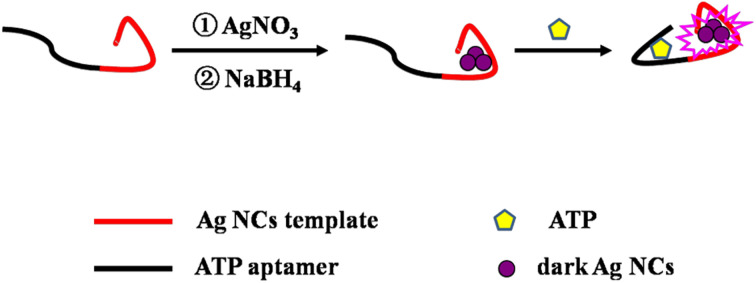

## 1 Introduction

Fluorescent metallic nanoclusters (*e.g.* AgNCs, AuNCs, and CuNCs), as ultra-small particles composed of several to about a hundred atoms, since they have sizes of less than 2 nm, the Fermi wavelength of electrons, but have properties like molecules, such as discrete electronic transitions and strong fluorescence,^1^ have gradually become popular fluorescence probes due to their good water dispersibility, size adjustability and unique optical properties.^2–5^ In particular, oligonucleotide-templated silver nanoclusters (DNA-AgNCs) possess more advantages, such as great biocompatibility, high photostability, hypotoxicity, ease of synthesis, adjustable light emission wavelength and large Stokes shift, than fluorophores or organic quantum dots.^6–8^ Therefore, they have been widely utilized to probe various analytes, including metal ions,^9,10^ enzymes,^11–13^ DNA,^14–16^ and biomolecules, in fluorescent biosensors.^17,18^

ATP, as a primary energy storage molecule, plays a key role in regulating biochemical pathways and intra-cellular metabolism in diverse tissues and organs^19^ and provides energy for various types of biochemical reaction in most living organisms.^20^ In addition, it has been used as a biomarker for some diseases, such as Alzheimer's disease, hypoxia,^21^ ischemia,^22^ Parkinson's disease,^23^ hypoglycemia,^24^ and some malignant tumors.^25^ Thus, in the past few decades, a lot of detection methods for ATP have been reported, such as fluorescence analysis,^26^ colorimetric methods,^27^ liquid chromatography,^28,29^ and electrochemical analysis.^30^ However, these methods inevitably suffer from time-consuming and laborious modification of electrodes and fluorescent probes and/or preparation of complex samples. In recent years, fluorescence detection methods based on distyrylbenzene (1) with two arms of dipicolylaminomethyl groups at the central benzene ring and its zinc complex (1-Zn),^26^ rhodamine derivatives,^31^ and labeled aptamer and magnetic nanoparticles^32^ have been applied to analyze ATP with good sensitivity and selectivity. However, the first two types of probes require complex organic synthesis, while the latter require 6-carboxyflurrescein labelling on the aptamer. These operations are not only complex but may also affect the detection effect. In addition, the biocompatibility of organic probes is poor. Hence, the development of a simple and sensitive detection strategy for ATP is urgently needed for biochemical studies, food hygiene applications and clinical diagnosis.

**Scheme 1:**
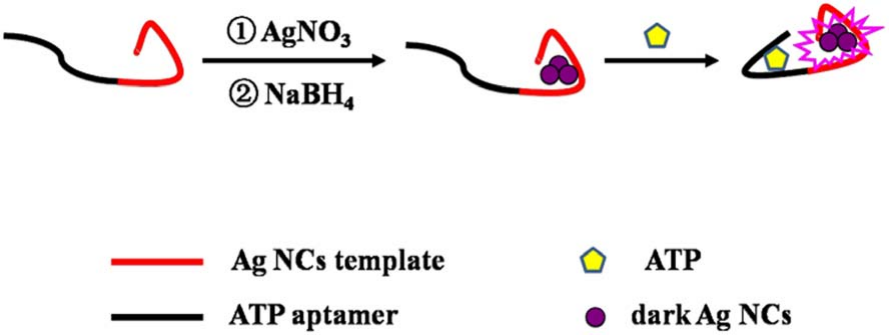
Schematic illustration of ATP detection utilizing DNA-templated AgNCs with aptamer.

Inspired by the significant increase in fluorescence when darkish DNA-AgNCs are placed together utilizing complementary linkers,^33^ a series of probes for the detection of ATP were constructed by our team based on this principle. These methods based on DNA-Ag NCs^34,35^ and DNA-Cu/Ag NCs^36^ have good sensitivity and selectivity and simple operation. However, LODs are not satisfactory at present. Hence, a novel strategy was constructed for the quantitative detection of ATP based on DNA-Ag NCs with an ATP aptamer, whose DNA template was designed based on a thesis from Zhang's team.^34^ Namely, two bases (TT or AA) are removed from the bases “TTTTT” or “AAAAA” of the DNA template^34^ so that they connect nucleation sequences and adapter sequences, respectively. Consequently, the LOD of the proposed probe becomes lower than that obtained without removing connecting base sequences. As shown in Scheme 1, the template contains an ATP aptamer lying on the left and an AgNC-nucleation sequence on the right. Darkish AgNCs on the right become significantly brighter in the presence of ATP owing to the conformational alteration of the ATP aptamer, leading to a change in the environment of AgNCs. Furthermore, a feasibility experiment was performed. As shown in ESI Fig. S1,† the fluorescence was obviously enhanced in the presence of 24 mM ATP. Thus, the proposed nanoprobe is not only simple, convenient and sensitive but also a green ATP detection assay.

## 2 Experimental section

### 2.1 Reagents and apparatus

Oligonucleotides, ATP, guanosine 3′-triphosphate (GTP), cytidine 3′-triphosphate (CTP), and uridine 3′-triphosphate (UTP) used in this study were purchased from Sangon Biotechnology Inc. (Shanghai, China), and the names and sequences of the DNA are listed in ESI Table S1.† Fetal bovine serum was provided by Yuanye Biotechnology Co. Ltd (Shanghai, China). Cu(NO_3_)_2_, Fe(NO_3_)_2_, Fe(NO_3_)_3_, Co(NO_3_)_2_, Mg(NO_3_)_2_, Ca(NO_3_)_2_, glucose (Glu), l-histidine (l-His), sodium borohydride (NaBH_4_, 98%) and silver nitrate (AgNO_3_, 99.8%) were obtained from Aladdin Bio-chem Technology Co. Ltd (Shanghai, China). All chemical reagents were of analytical grade and used without further purification. Phosphate buffer solution (PBS, 20 mM, pH 7.0) was used in all experiments. All solutions were prepared using Milli-Q water (18.2 MΩ cm).

Fluorescence measurements were recorded with an Edinburgh Instruments FS5 fluorescence spectrophotometer at room temperature, and slit widths were 2.0 nm and 4.0 nm for excitation and emission, respectively. The morphologies and average sizes of AgNCs were studied using a JEOL JEM-2100 transmission electron microscope with an acceleration voltage of 200 kV. Time-resolved fluorescence measurements were performed using an FS5 fluorescent lifetime spectrometer (Edinburgh Instruments, Livingston, UK) operating in the time-correlated single photon counting (TCSPC) mode using a semiconductor laser (405 nm) as the excitation source. Commercial software from Edinburgh Instruments was used for data analyses. When 
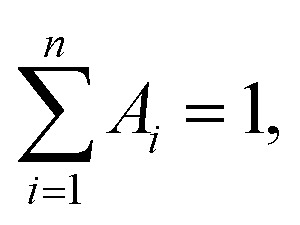
 the average excited state lifetime is expressed by the equation 
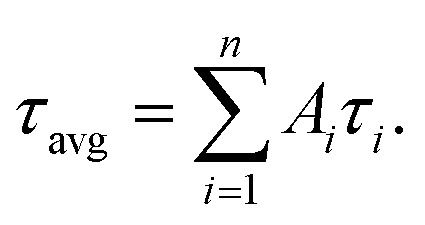
 The reported spectrum of each sample represents the average of three scans. X-ray photoelectron spectroscopy (XPS) (ESCALAB 220i-XL, VG Scientific, England) was performed using monochromic Al Kα as the source at 1486.6 eV.

### 2.2 Synthesis of Ag NCs

DNA-AgNCs were synthesized according to the procedure previously reported in the literature.^36^ Briefly, Ag NO_3_ (18 μM) and DNA (3.0 μM) were sequentially added to PBS solution (20 mM, pH 7) under uniform stirring. The mixture was incubated in the dark at 4 °C for 20 min, followed by the addition of fresh NaBH_4_ (18 μM) and stirred for 1 min; then, the mixture was kept away from light for 1 h at 4 °C. After 1 h, the reduced DNA-AgNC solution was stored at 4 °C for the ATP assay.

### 2.3 Detection of ATP

ATP (0–39 mM) of different concentrations were sequentially added into the abovementioned DNA-AgNC solution. The fluorescence spectrum measurements were performed at room temperature. For the assay of the specificity of the proposed probe, ATP, and ATP analogues, including GTP, UTP, and CTP, were measured under same conditions as ATP. Additionally, other interfering agents, including Cu^2+^, Fe^2+^, Fe^3+^, Co^2+^, Mg^2+^, Ca^2+^, Glu and l-His, were investigated.

### 2.4 Application of the proposed nanoprobe

To further investigate the practicability of the proposed nanoprobe, ATP in fetal bovine serum solution which was diluted 100 times was analyzed. The samples were spiked with ATP of varying different concentrations and measured by employing the same method as for ATP detection.

### 2.5 Method of quantum yield measurement and calculation

The measurement of absolute photoluminescence quantum yield (APLQY) in this study used the SC-30 Integrating Sphere Module of an FS5 Sample Module, diffuse reflectance spectra and absorbance spectra based on diffuse reflectance. The absolute quantum yield, *η*, is the ratio of the number of photons emitted to the number of photons absorbed: *η* = *N*^em^/*N*^abs^. It was calculated using “direct excitation” measurements where one records the scatter and emission of a sample being directly excited by the radiation from the excitation monochromator alone.

## 3 Results and discussion

### 3.1 Optical characterization of DNA-Ag NCs

**Fig. 1:**
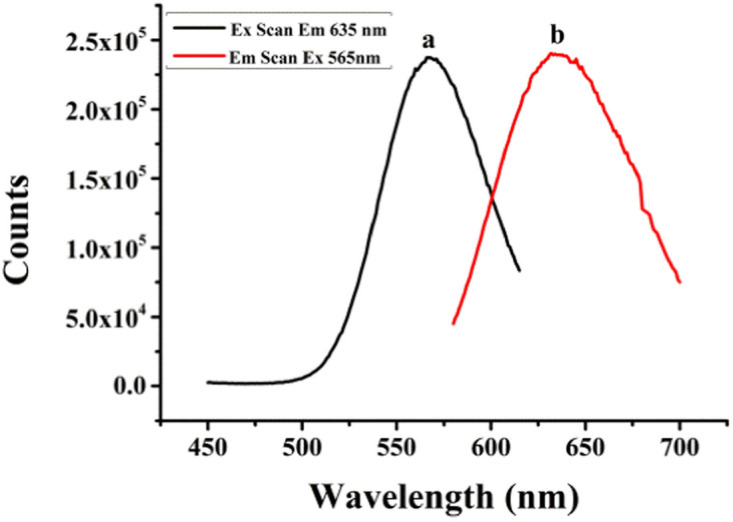
Excitation (a) and emission (b) spectra of (L)BT3A3-AgNCs.

It has been reported that the optical properties of AgNCs are determined by different DNA templates, which depend on the characteristic of their base sequences and secondary structures.^37,38^ Therefore, templates of DNA named (L)BT3A3, BT3A3 and BT3A3(R) were designed. ESI Table S1† lists their sequences. As shown in ESI Table S1,† the aptamer of ATP is located in the middle part (italic) of the DNA, while the C-rich sequence stabilized AgNCs lie at 5′ and/or 3′ ends (bold). BT3A3(R) and (L)BT3A3 come from BT3A3, from which right (or left) C-rich segments and AAA (or TTT) linkers (underlined) of BT3A3 have been removed. Fig. 1 shows the optical characterization of (L)BT3A3-AgNCs. Excitation (curve a) and emission (curve b) peaks are at 565 and 635 nm, respectively. Similarly, the excitation and emission spectra of BT3A3 and BT3A3(R)-AgNCs were also studied, and it can be seen from ESI Fig. S2A and B† that excitation and emission wavelengths are 560 and 640 nm for BT3A3-Ag NCs and 565 and 650 nm for BT3A53(R)-AgNCs. In addition, the UV-Vis absorption spectra of (L)BT3A3-AgNCs were collected for different concentrations of ATP. Two peaks appear in every curve of ESI Fig. S3† and 430 nm peaks and 565 nm peaks should be attributed to the characteristic plasmon absorption band of Ag nanoparticles and the absorption band of AgNCs, respectively.^35^ Therefore, Ag nanoparticles exist in (L)BT3A3-AgNCs. The absorption gradually increases with increasing ATP concentration, demonstrating the amount of enhancement of (L)BT3A3-AgNCs.

In addition, the images of the probe before and after exposure to UV light (*λ*_max_ = 365 nm) were taken. As shown in ESI Fig. S4A,† it looks colourless (sample 0 in ESI Fig. S4A†) before exposure to UV light and looks light red (sample 1 in ESI Fig. S4A†) after exposure to UV light. This indicates the emission of red light under irradiation by UV light. Similarly, the images of the probe were measured before and after the addition of 10 mM ATP under illumination by UV light. As shown in ESI Fig. S4B,† the images of the probe upon the addition of 10 mM ATP show light red (sample 1 in ESI Fig. S4B†), while others with 0 mM ATP look colourless (sample 0 in ESI Fig. S4B†). Hence, the color of the probe changed after adding ATP, indicating that ATP specifically binds to its adapter.

The fluorescence intensity of each DNA-AgNC against storage time was detected since the stability of the probe has a great effect on its detection performance. As shown in ESI Fig. S5,† the fluorescence intensity of (L)BT3A3-AgNCs reaches a plateau after 3 h, then remains almost unchanged for 1.5 h. However, the fluorescence intensity of BT3A3-AgNCs declines with storage time. The fluorescence intensity of BT3A3(R)-AgNCs gradually increases, then declines. The changes in fluorescence intensity of (L)BT3A3-AgNCs and BT3A3(R)-AgNCs are similar, as their nucleation sequences are located at one end of the DNA template. The changes in fluorescence intensity of BT3A3-AgNCs are different from them, as its nucleation sequence is located at both ends of the DNA template. It can be seen that the stability of (L)BT3A3-AgNCs is better than those of others.

The quantum yields (QY) of (L)BT3A3-AgNC, BT3A3-AgNC, and BT3A3(R)-AgNC probes were measured to evaluate the ability of these probes to emit fluorescence. As displayed in ESI Fig. S6,† they are 28.28%, 43.07%, and 29.48%, respectively. Apparently, the highest quantum yield of BT3A3-AgNCs is consistent with its two rich-C sequences, and the quantum yields of (L)BT3A3-AgNCs and BT3A3 (R)-AgNCs are similar, owing to them only having a rich-C sequence.

### 3.2 Characteristics of (L)BT3A3-Ag NCs

**Fig. 2:**
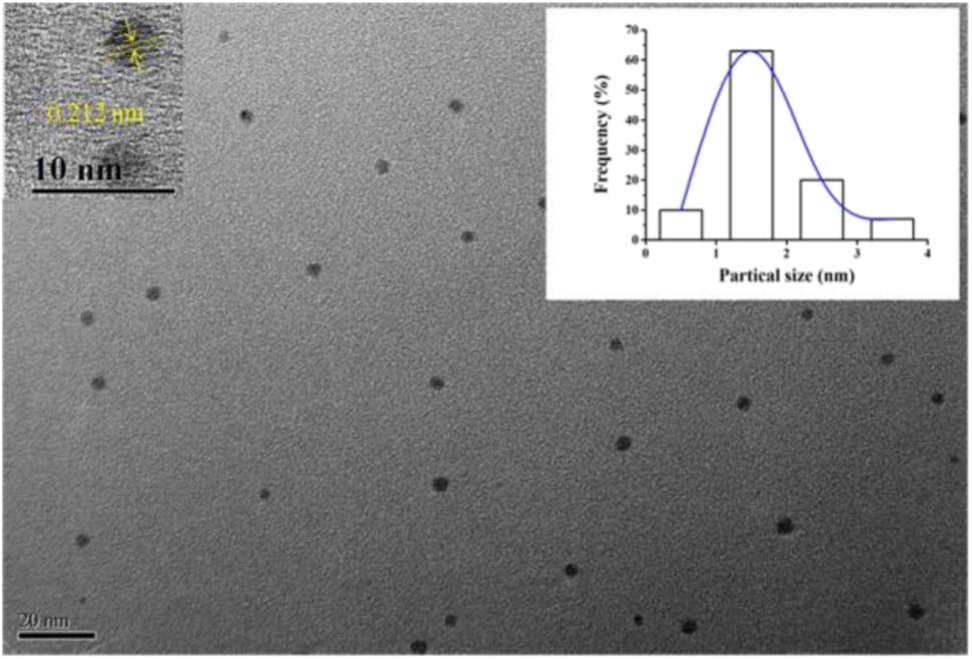
TEM image of (L)BT3A3-Ag NCs (inset: HRTEM image and size distribution histogram).

The morphologies and size of (L)BT3A3-AgNCs were studied using transmission electron microscopy. (L)BT3A3-AgNCs are distributed uniformly in Fig. 2. The average diameter of about 2 nm for proposed DNA-AgNCs is in accordance with the size of metal NCs being less than 2 nm (ref. 39) and was obtained from a histogram fitted using the Lorentzian function. Additionally, HRTEM experiments were carried out. The obvious crystal lattice structures of AgNCs are presented in the inset of Fig. 2, which reveals their high crystallinities, where the lattice spacing is 0.212 nm.

**Fig. 3:**
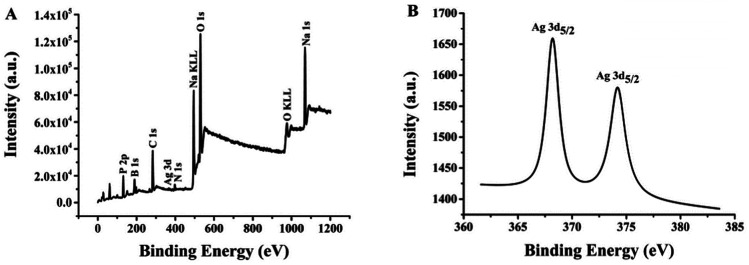
(A) XPS spectrum of (L)BT3A3-AgNCs. (B) Ag 3d region of the XPS spectrum of (L)BT3A3-AgNCs.

Ag 3d spectra and XPS wide scan survey spectra were performed to confirm the elemental and valence states of Ag element in (L)BT3A3-AgNCs. Nine peaks in Fig. 3A corroborate the presence of P, B, C, Ag, N, Na and O elements. As shown in Fig. 3B, 2 peaks appear in the expanded spectrum of Ag 3d, and the binding energies of 368.2 eV for Ag 3d_5/2_ and 374.2 eV for Ag 3d_3/2_ could be assigned to Ag(1) and Ag(0) in (L)BT3A3-Ag NCs,^40,41^ respectively. Hence, (L)BT3A3-Ag NCs are compounds of Ag(1) and Ag(0).

### 3.3 Optimization of experimental conditions

#### 3.3.1 Responses of different DNA-Ag NCs to ATP

AgNCs synthesized from different DNA templates have different effects on ATP detection due to the different base sequences and secondary structures of DNA templates.^37,38^ The fluorescence of (L)BT3A3-AgNCs, BT3A3-AgNCs and BT3A3(R)-AgNCs upon the addition of 21 mM ATP was determined. ESI Fig. S7† shows that the fluorescence of all three immediately increases when ATP is added, but the relative fluorescence intensity (*F*/*F*_0_, where *F* and *F*_0_ are the fluorescence intensities of AgNCs after adding 21 mM ATP and no ATP, respectively) of (L)BT3A3-AgNCs is most obviously enhanced among them, when more than 50-fold fluorescence enhancement was observed. Therefore, (L)BT3A3-AgNCs was chosen as the best candidate for following experiments. Furthermore, to confirm whether ATP interacts with AgNCs, experiments were carried out in which 10 mM ATP was added to (L)BT3A3-AgNC and C-DNA-AgNC (where C-DNA sequences are the same as the nucleation sequences of (L)BT3A3) solutions, respectively. As shown as in ESI Fig. S8,† the fluorescence of C-DNA-Ag NCs increased a little in the presence of 10 mM ATP. However, the fluorescence of (L)BT3A3-AgNCs was very obviously enhanced. Thus, ATP mainly binds with its aptamer instead of AgNCs owing to the ATP aptamer specifically binding to its target.

#### 3.3.2 Determination of the reaction time of (L)BT3A3-AgNCs with ATP

The reaction time of (L)BT3A3-AgNCs with 15 mM ATP was determined to improve the sensitivity of the proposed probe for the detection of ATP. ESI Fig. S9† demonstrates that the reaction time is 5 min. Therefore, 5 min is the optimal reaction time for the nanoprobe to detect ATP.

#### 3.3.3 Optimization of pH

The value of pH greatly affects the accurate and quantitative detection of ATP. Therefore, the fluorescence intensity of (L)BT3A3-AgNCs was studied at pH 5, 6, 7, 8, and 9. As shown in ESI Fig. S10,† the relative fluorescence intensity (*F*/*F*_0_, where *F* and *F*_0_ are the fluorescence intensities of AgNCs after adding 12 mM ATP and no ATP, respectively) of (L)BT3A3-AgNCs show an increasing trend from pH 5 to 7, reaching its peak at pH 7 and decreasing from pH 7 to 9. Thus, all experiments were conducted using pH 7.

### 3.4 Assay of ATP

**Fig. 4:**
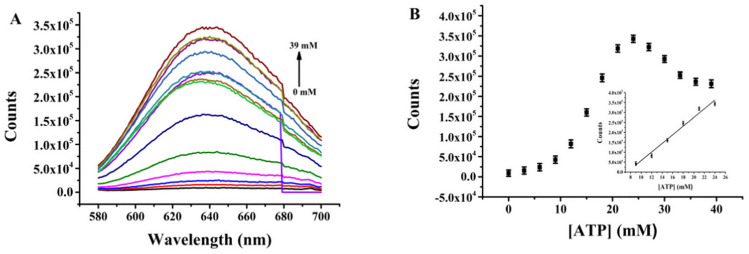
(A) Fluorescence emission spectra of (L)BT3A3-AgNCs in the presence of ATP (0–39 mM). (B) ATP concentration-dependent changes in fluorescence intensity at 635 nm. (Inset) Linear fit between *F*_635nm_ and ATP concentration (9–24 mM). Error bars represent the standard deviation of three repeated experiments.

Quantitative determination of ATP exploiting the proposed nanoprobe was performed under optimal conditions. The fluorescence intensity of (L)BT3A3-AgNCs was recorded with different concentrations of ATP. Fig. 4A and B depict that the fluorescence intensity continues to increase when ATP concentration increases, with a good linear relationship within the range of 9–24 mM (*R*^2^ = 0.9755, where the linear regression equation is *F* = −162507.3 + 21888.6*C*_ATP_) and a limit of detection (LOD) of 3.0 μM, which was calculated according to 3*σ*_0_/*k* (where the value of *σ*_0_ is obtained from the standard deviation of the background, and *k* represents the slope of the calibration line). ESI Table S2† shows that the LOD of the proposed nanoprobe is superior to that of previous sensors for ATP.^35,36,42,43^ Therefore, the proposed nanoprobe has high sensitivity.

To investigate the quenching mechanism of fluorescence in (L)BT3A3-AgNCs, the lifetimes of (L)BT3A3-AgNCs were studied at different concentrations of ATP with an emission wavelength of 635 nm (ESI Fig. S11†). The fluorescence transients of (L)BT3A3-AgNCs exhibit haploid exponential time constants (ESI Table S3†) with an average lifetime of 3.26 ns. The lifetimes of (L)BT3A3-AgNCs hardly change as the ATP concentration increases, therefore, showing that the interaction between (L)BT3A3-AgNCs and ATP is a static process.

### 3.5 Selectivity of the nanoprobe toward ATP

**Fig. 5:**
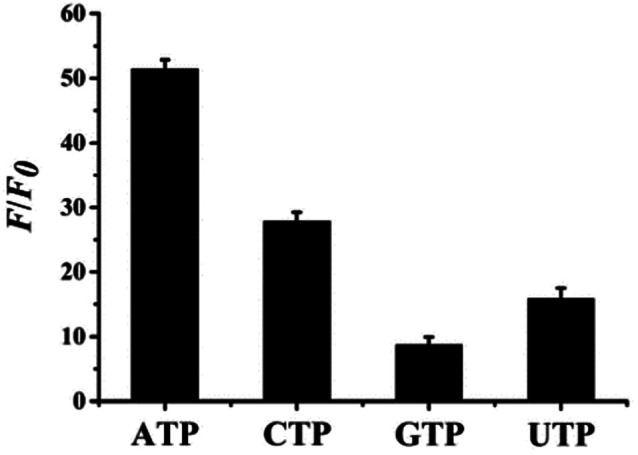
Selectivity of the ATP detection system. The concentrations of ATP, CTP, UTP and GTP are 21 mM, respectively. Error bars represent the standard deviation of three repeated experiments.

Selectivity is one of the most important parameters for a fluorescence probe with good properties therefore, ATP and other nucleotide analogues, including CTP, UTP and GTP, were investigated at concentrations of 21 mM. As shown in Fig. 5, relative fluorescences (*F*/*F*_0_) are 51.4, 27.9, 8.73, and 15.9 in the presence of ATP, CTP, GTP, and UTP. Therefore, the relative fluorescence in the presence of ATP increases to a maximum, due to the high specific binding ability of ATP and its aptamers.^44^ While other nucleotide analogs cause a certain degree of increase, and *F*/*F*_0_ even reaches 27.9 for CTP. Conversely, the fluorescence response of the proposed probe towards ATP was monitored in the presence of interfering analytes, including CTP, UTP, and GTP, as shown in ESI Fig. S12.† The black bars represent the relative fluorescent intensity (*F*/*F*_0_) of (L)BT3A3-Ag NCs in the presence 10 mM ATP, and the red bars represent *F*/*F*_0_ of (L)BT3A3-AgNCs when 10 mM ATP and 5 mM ATP, UTP, CTP, and GTP. It exhibits significant fluorescence enhancement towards ATP. Although the fluorescence also increases a little towards other interference analytes, it is not obvious. CTP increases more than UTP and GTP, which is consistent with above-mentioned experimental results. Therefore, the selectivity of the proposed probe needs future improvement for the assay of ATP. In addition, other interfering agents, including Cu^2+^, Fe^2+^, Fe^3+^, Co^2+^, Mg^2+^, Ca^2+^, Glu and l-His, were investigated for the evaluation of the specificity of the proposed nanoprobe. As shown in ESI Fig. S13,† Cu^2+^, Fe^2+^, Fe^3+^ and Co^2+^ hardly cause any change in the relative fluorescent intensity (*F*/*F*_0_). *F*/*F*_0_ for Mg^2+^ and Ca^2+^ are less than 1 and for Glu and l-His are not more than 0.4. The transitional metallic elements, Cu^2+^, Fe^2+^, Fe^3+^ and Co^2+^, can quench AgNCs through energy transfer or electron transfer,^45^ while the influence on AgNCs of Mg^2+^, Ca^2+^, Glu and l-His can be neglected.

### 3.6 Application of the proposed nanoprobe

To evaluate the practicability of the proposed nanoprobe, (L)BT3A3-AgNCs were exploited to determine ATP spiked in a 100-fold diluted fetal bovine serum. As presented in Table 1, recoveries used this strategy are between 99.2% and 104.3%. Moreover, relative standard deviations are in the range of 1.09 and 1.92, which are comparable with the value previously reported.^46,47^ Therefore, results demonstrate that the nanoprobe not only has reliability but also good precision for determining ATP in fetal bovine serum.

**Table 1:** Determination of ATP in diluted fetal bovine serum solution (*N* = 3)

Samples	Spiked (mM)	Measured (mM)	Recovery (%)	RSD (%)
		Mean^a^ ± SD^b^		
1	7	7.3 ± 0.14	104.3	1.92
2	10	10.1 ± 0.11	101.0	1.09
3	13	12.9 ± 0.15	99.2	1.16
4	16	16.3 ± 0.18	101.9	1.10

a Mean of three determinations.

b SD = standard deviation. RSD = relative standard deviation.

## 4 Conclusions

In conclusion, a turn-on fluorescence nanoprobe has been developed for ATP detection based on DNA-AgNCs. It has a lot of advantages, including simplicity, sensitivity, specificity, hypotoxicity, low cost, and the avoidance of chemical modification. Furthermore, this proposed nanoprobe succeeded in determining ATP in diluted fetal bovine serum. Therefore, the proposed probe has great potential in clinical diagnosis and biological fields.

## Conflicts of interest

There are no conflicts to declare.

## Supplementary Material

RA-014-D3RA07077H-s001
