# Supplementary material for: Turn-on fluorescent nanoprobe for ATP detection based on DNA-templated silver nanoclusters
Source: RSC Adv. 2024 Feb 13;14(8):5594–9. doi: 10.1039/d3ra07077h (PMC10863603; doi:10.1039/d3ra07077h)
Supplement: RA-014-D3RA07077H-s001 [file RA-014-D3RA07077H-s001.pdf]

Electronic Supplementary Material (ESI) for RSC Advances.
This journal is © The Royal Society of Chemistry 2024

Electronic Supplementary Information

A turn-on fluorescent nanoprobe for ATP detection based on DNA- templated silver nanoclusters

Yuxia Li, Zeting Meng, Yating Liu, Baozhu Zhang*

Department of Chemistry and Chemical Engineering, Jinzhong University, Yuci 030619, P. R. China

---

*Corresponding author.

E-mail address: zhangbaozhu518@126.com

Table S1 Names and sequences of the oligonucleotides.

Oligonucleotids	Sequences (5´- 3´)
C-DNA	**CCCTAACTCCCC**
(L)BT3A3	*AACCTGGGGGAGTATTGCGGAGGAAGGT*AAA**CCCTAACTCCCC**
BT3A3(R)	**CCCTTAATCCCC**TTT*AACCTGGGGGAGTATTGCGGAGGAAGGT*
BT3A3	**CCCTTAATCCCC**TTT*AACCTGGGGGAGTATTGCGGAGGAAGGT* AAA**CCCTAACTCCCC**

Table S2 Comparison of different methods for the detection of ATP.

Detection methods	LOD	Linear range	References
Fluorescence DNA-Cu/Ag NCs	7.0 µM	2-18 mM	36
DNA-Ag NC fluorescence light-up system	0.44 mM	0-4 mM	35
Fluorescence-based core–shell Ag@SiO_2 nanoflares	8 µM	0-500 µM	42
Light-up DNA-scaffolded silver nanoclusters	0.81 mM	1-6 mM	43
Fluorescence DNA-Ag NCs	3.0 µM	6-24 mM	this work

Table S3 The lifetimes of (L)BT3A3-Ag NCs in the absence and presence of ATP.

Samples	[ATP](mM)	τ(ns)	χ^2
(L)BT3A3-Ag NCs	0	3.32	1.1977
(L)BT3A3-Ag NCs + ATP	5	3.34	1.1178
	10	3.29	1.1090
	15	3.23	1.1708
	20	3.11	1.0973

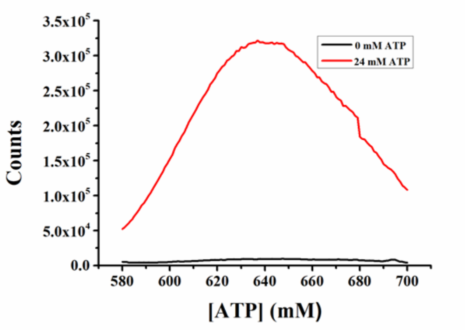

Fig. S1 Feasibility for the detection of ATP.

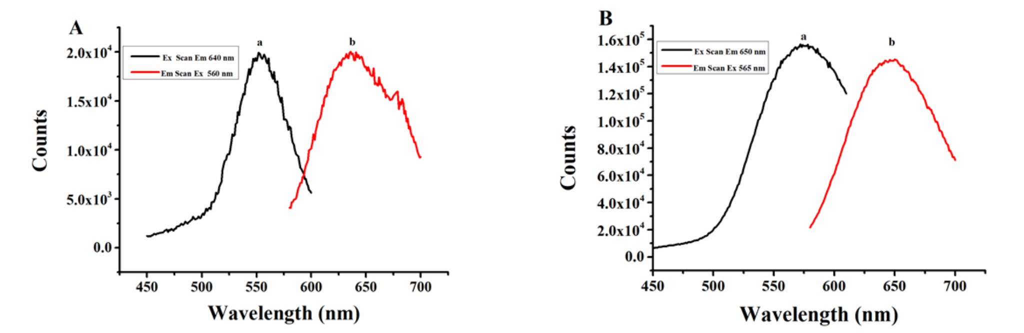

Fig. S2 (A) The excitation (a) and emission (b) spectra of BT3A3-Ag NCs. (B) The excitation (a) and emission (b) spectra of BT3A3(R)-Ag NCs.

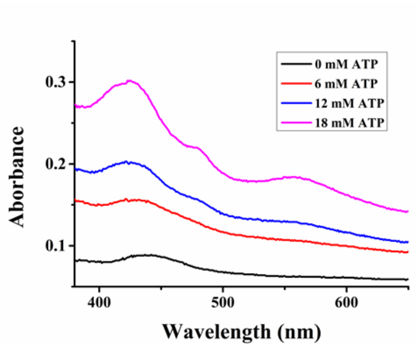

Fig. S3 UV-Vis absorption spectra of (L)BT3A3-Ag NCs in the absence and presence of ATP

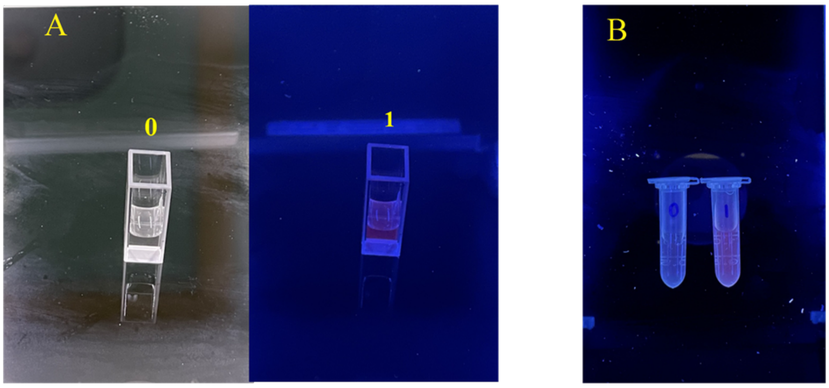

Fig. S4 (A)**The** images of (L)BT3A3-Ag NCs before (0) and under (1) illumination of UV light (λ_{max} = 365 nm). (B)The image of (L)BT3A3-Ag NCs before (0) and after (1) the addition of 10 mM ATP under illumination of UV light (λ_{max} = 365 nm).

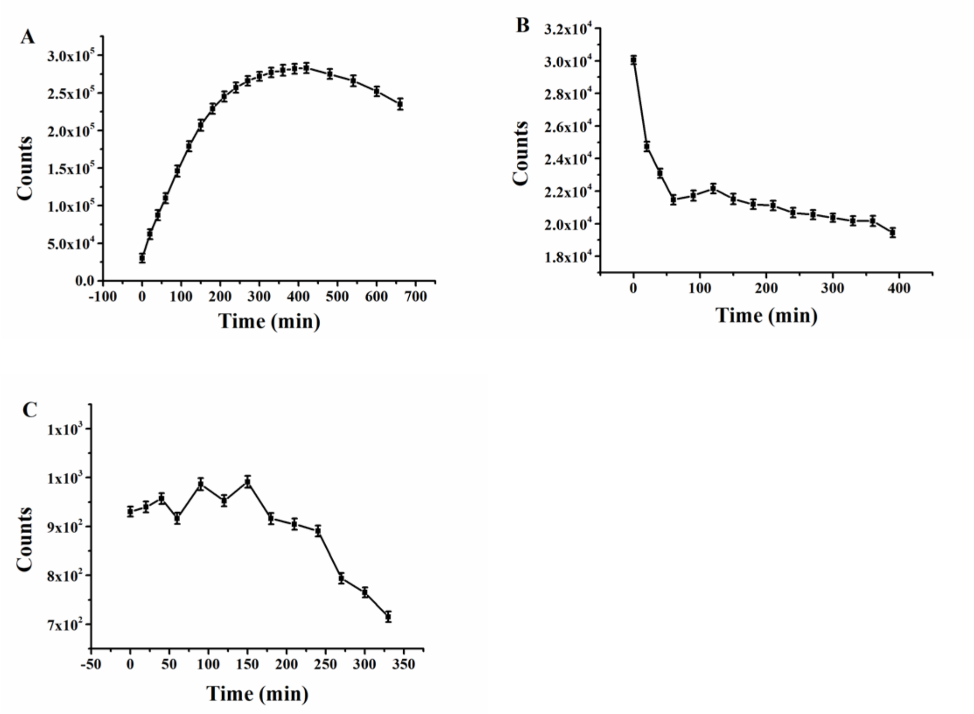

Fig. S5 The change of fluorescence intensity of (L)BT3A3-Ag NCs (A), BT3A3-Ag NCs (B), and BT3A3(R)-Ag NCs(C), *against* the increasing time. Error bars represent the standard deviation of three independent measurements. c(DNA) = 3.0 μM.

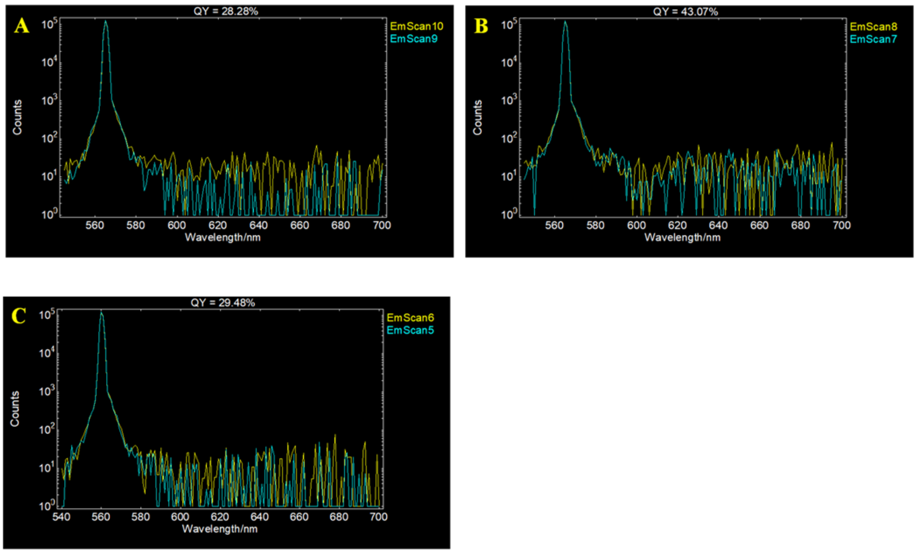

Fig. S6 The quantum yield (QY) of (L)BT3A3-AgNCs (λ_{ex} = 565 nm, integrated wavelength range:545-700 nm) (A), BT3A3-AgNCs (λ_{ex} = 560 nm, integrated wavelength range:540-700 nm) (B), and BT3A3(R)-AgNCs (λ_{ex} = 565 nm, integrated wavelength range:545-700 nm) (C) probes.

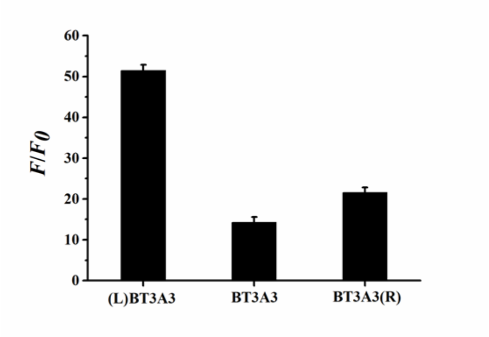

Fig. S7 The relative fluorescence intensity (F/F_0) of different DNA-AgNCs. F_0 and F are the emission intensity of the DNA-Ag NCs before and after the addition of 21 mM ATP, respectively. The error bars represent the standard deviation of three independent measurements.

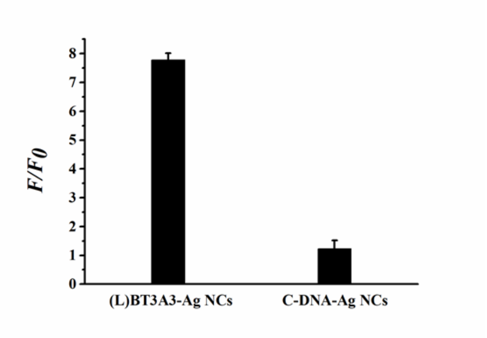

Fig. S8 The relative fluorescence intensity (F/F_0) of different DNA-AgNCs. F_0 and F are the emission intensity of the DNA-Ag NCs before and after the addition of 10 mM ATP, respectively. The error bars represent the standard deviation of three independent measurements.

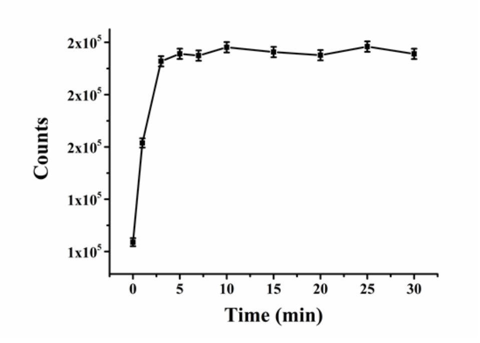

Fig. S9 The fluorescence intensity of (L)BT3A3-AgNCs with 15 mM ATP against the reaction time.

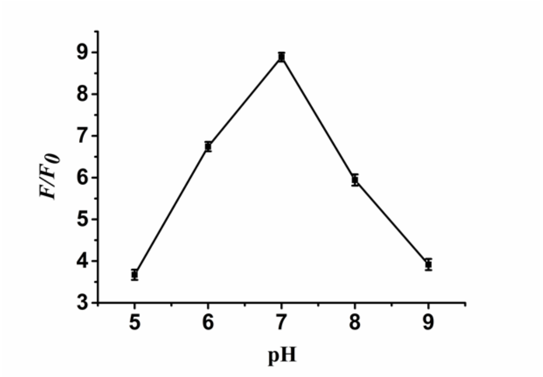

Fig. S10 Relative fluorescence intensity (F/F_0) of (L)BT3T3-Ag NCs at different pH values. F_0 and F are the maximum emission intensity of (L)BT3T3-Ag NCs without and with 12 mM ATP, respectively. The error bars represent the standard deviation of three independent measurements.

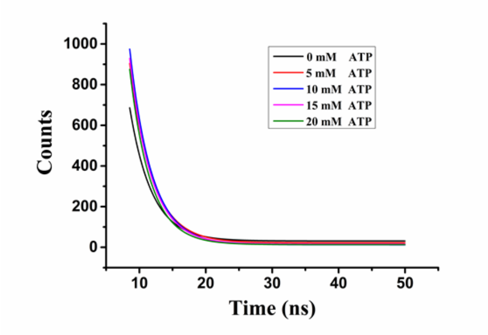

Fig. S11 The fluorescence lifetimes of (L)BT3A3-Ag NCs (excitation at 405 nm and emission at 635 nm) in the absence and presence of ATP.

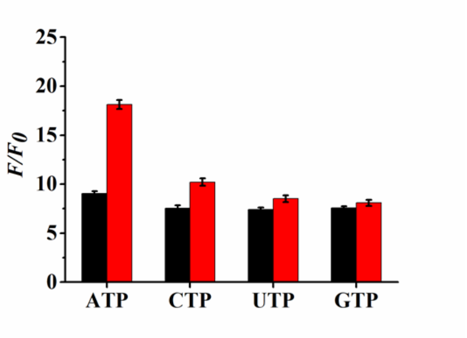

Fig. S12 Selectivity of the ATP detection system. The relative fluorescent intensity (F/F_0) of (L)BT3A3-Ag NCs in the presence of ATP (10 mM, black bars) and coexistence (red bars) of ATP (10 mM) and ATP, CTP, UTP, and GTP (5 mM).

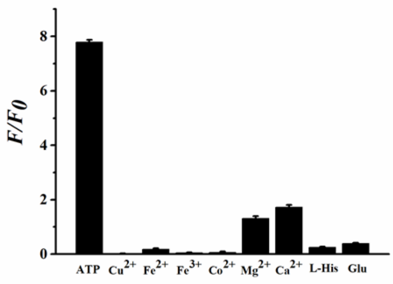

Fig. S13 Selectivity of the ATP detection system. The concentrations of ATP, Cu^{2+}, Fe^{2+}, Fe^{3+}, Co^{2+}, Mg^{2+}, Ca^{2+}, Glucose (Glu) and L-Histidine (L-His) are 10 mM, respectively. Error bars represent the standard deviation of three repetitive experiments.
